# Blood biomarker profiles and exceptional longevity: comparison of centenarians and non-centenarians in a 35-year follow-up of the Swedish AMORIS cohort

**DOI:** 10.1007/s11357-023-00936-w

**Published:** 2023-09-19

**Authors:** Shunsuke Murata, Marcus Ebeling, Anna C. Meyer, Katharina Schmidt-Mende, Niklas Hammar, Karin Modig

**Affiliations:** 1 Unit of epidemiology, Institute of Environmental Medicine, Karolinska Institutet, Box 210, 17177 Stockholm, Sweden; 2 Department of Preventive Medicine and Epidemiology, National Cerebral and Cardiovascular Center, Suita, Japan; 3 Laboratory of Population Health, Max Planck Institute for Demographic Research, Rostock, Germany; 4 Academic Primary Health Care Centre, Stockholm Region, Stockholm, Sweden; 5 Division of Family Medicine and Primary Care, Department of Neurobiology, Care Sciences and Society, Karolinska Institutet, Huddinge, Sweden

**Keywords:** Centenarians, Biomarkers, Longevity, Homogeneity

## Abstract

Comparing biomarker profiles measured at similar ages, but earlier in life, among exceptionally long-lived individuals and their shorter-lived peers can improve our understanding of aging processes. This study aimed to (i) describe and compare biomarker profiles at similar ages between 64 and 99 among individuals eventually becoming centenarians and their shorter-lived peers, (ii) investigate the association between specific biomarker values and the chance of reaching age 100, and (iii) examine to what extent centenarians have homogenous biomarker profiles earlier in life. Participants in the population-based AMORIS cohort with information on blood-based biomarkers measured during 1985–1996 were followed in Swedish register data for up to 35 years. We examined biomarkers of metabolism, inflammation, liver, renal, anemia, and nutritional status using descriptive statistics, logistic regression, and cluster analysis. In total, 1224 participants (84.6% females) lived to their 100th birthday. Higher levels of total cholesterol and iron and lower levels of glucose, creatinine, uric acid, aspartate aminotransferase, gamma-glutamyl transferase, alkaline phosphatase, lactate dehydrogenase, and total iron-binding capacity were associated with reaching 100 years. Centenarians overall displayed rather homogenous biomarker profiles. Already from age 65 and onwards, centenarians displayed more favorable biomarker values in commonly available biomarkers than individuals dying before age 100. The differences in biomarker values between centenarians and non-centenarians more than one decade prior death suggest that genetic and/or possibly modifiable lifestyle factors reflected in these biomarker levels may play an important role for exceptional longevity.

**Supplementary Information:**

The online version contains supplementary material available at 10.1007/s11357-023-00936-w.

## Introduction

The global number of centenarians—individuals who survive at least to their 100th birthday—has roughly doubled every decade since 1950 and is projected to quintuple between 2022 and 2050 [1, 2]. Exceptional longevity is the result of a complex interplay of several determinants, which is yet poorly understood and includes both genetic predisposition and lifestyle factors [3]. Studying centenarians and exploring differences between them and their shorter-lived peers provides an opportunity to improve our understanding of how aging processes unfold and exceptionally long survival is promoted.

Despite the claim that chance plays an important role in the achievement of exceptional longevity, it has repeatedly been shown that already earlier in life, centenarians are a selected group with fewer disabilities, comorbidities, hospitalizations, and better cognitive function compared to non-centenarians [4–6]. While the cited studies focus on specific health outcomes, blood-based biomarkers can provide additional information about health status already before other observable outcomes occur. A Japanese cohort study found that low inflammation defined by cytomegalovirus titer, interleukin-6, tumor necrosis factor-alpha, and C-reactive protein (CRP) was an important predictor for exceptional survival [7]. Improved survival in old age has also been linked to lower creatinine, higher albumin, and several circulating biomarkers (N-terminal pro-B-type natriuretic peptide, interleukin-6, cystatin C, and cholinesterase) [8]. Cross-sectional studies have found centenarians to have lower total cholesterol [9] and insulin tolerance [10] than younger elderly. However, since biomarkers change with age, it is difficult to draw conclusions from cross-sectional studies that compare samples drawn at different ages.

Knowledge of how centenarians’ biomarker profiles differ from those of non-centenarians at comparable ages already earlier in life is scarce. The lack of suitable, large prospective data with long follow-up is one likely reason for this. The Japanese cohort mentioned above included individuals aged 85+ only, and more than half of them were already centenarians at baseline enrollment. Since health selection likely starts even earlier than age 85, it is important to examine potential differences between long-lived individuals and those with average life spans already several years before—or during the process of—health deterioration.

Moreover, several studies have reported that centenarians are not such a homogeneous population as sometimes perceived. An Italian study based on 602 centenarians identified three subgroups with distinct health profiles [11]. It was found that 20% of the centenarians were in good health, 33% had intermediate health status, and 47% were in poor health. A Danish study also detected three distinct subgroups defined by health status: robust, intermediate, and frail centenarians [12]. About half of the Danish centenarians were in the “robust” group. A German study using health insurance data from 1121 centenarians found four distinct comorbidity profiles, and only a small proportion of centenarians had a low morbidity burden [13]. These findings raise the question of whether such heterogeneity in centenarians’ health profiles is already visible earlier in life and, for example, reflected in their biomarker profiles. Uncovering potential heterogeneity in such profiles more than one decade ago may help us understand characteristics of health trajectories associated with exceptional longevity.

The AMORIS (Apolipoprotein MOrtality RISk) cohort offers a unique opportunity to compare biomarkers measured at similar ages but earlier in life between centenarians and their shorter-lived peers. The cohort contains a variety of biomarkers assessed approximately 30 years ago and was linked to several administrative health registers with data until 2020. Using these data, we aim to (i) describe biomarker profiles earlier in life among individuals eventually becoming centenarians and their shorter-lived peers, (ii) investigate the association between a set of biomarkers and the chance of reaching age 100 with up to 35 years of follow-up, and (iii) investigate differences in biomarker profiles within the centenarian population.

## Methods

### Data sources and study population

The population-based AMORIS cohort consists of all individuals who underwent clinical laboratory testing at the Central Automation Laboratories, either as part of routine general health checkups or as outpatients referred for laboratory testing, between 1985 and 1996 in Stockholm County, which applies to more than 800,000 individuals. The cohort has been described in detail elsewhere [14, 15]. All laboratory analyses were performed using fully automated procedures on fresh blood samples, employing a consistent and well-documented methodology [14, 15]. Several Swedish registers have been linked to the AMORIS cohort through the unique Swedish personal identification number enabling longitudinal follow-up of the participants until the end of 2020. In this study, the National Patient Register was used to retrieve information on disease diagnoses, the Cause of Death Registry to identify the date of death, and the Total Population Registry to ensure individuals were alive and residing in Sweden. Charlson Comorbidity Index (CCI) was calculated based on hospitalizations recorded in the National Patient Register 10 years prior to the date of the first blood sample [16]. Detailed diagnose codes (ICD 8 and 9) and weighting of specific diagnoses were based on a previous study with publicly available script [16].

Birth cohorts born between 1893 to 1920 were included, enabling follow-up of all participants until age 100. Individuals were 64 to 99 years old at the time of their blood measurement. Individuals who emigrated during the follow-up were excluded (*n*=247). The final study population consisted of 44,636 participants followed from their first blood measurement until their date of death. Of these, 1224 individuals (2.7%) reached their 100th birthday, comprising the centenarian population. This proportion is very similar to the chance of reaching 100 in the general population of Stockholm in the same time period.

The study was approved by the Stockholm regional ethical review board (reference number 2018/2401-31). The ethical board waived the need for informed consent due to the size of the cohort and the fact that many of the participants had already died.

### Biomarker measurement

Twelve blood-based biomarkers related to inflammation and metabolic, liver, and kidney function as well as potential malnutrition and anemia were included, all of which have been associated with aging or mortality in previous studies (supplemental table 1) [8, 17–19]. The biomarker related to inflammation was uric acid; total cholesterol (TC) and glucose to metabolic status/function; alanine aminotransferase (ALAT), aspartate aminotransferase (ASAT), albumin, gamma-glutamyl transferase (GGT), alkaline phosphatase (ALP), and lactate dehydrogenase (LD) to liver function; creatinine to kidney functioning; iron and total iron-binding capacity (TIBC) to anemia; and albumin to nutrition. The first measurement of each biomarker was used. For individuals with missing values on some biomarkers (see supplemental table 2 for more information on missingness), we decided to impute these values since complete case analysis (excluding participants with missing values) can lead to selection bias. Missing values were imputed using multiple imputation. Detailed methods of multiple imputation are explained in the supplemental materials. A comparison of imputed and complete case data is shown in supplemental table 2 and 3. Analyses were additionally run for complete-case data and are shown in the supplemental materials as sensitivity analyses.

### Statistical analysis

In the first step, we investigated the distributions of biomarker values between centenarians and non-centenarians by estimating the 10th, 25th, 50th, 75th, and 90th quantiles of the respective distribution. Note that results additionally stratifying non-centenarians by age at death are included in the supplemental materials, as well as results from quantile regressions indicating if the quantiles are statistically different.

In the second step, we investigated the associations between each biomarker and the likelihood of becoming a centenarian. Logistic regression models were fitted separately for each biomarker. In these models, biomarkers were categorized into five groups (very low, low-medium, medium, high-medium, and very high) based on the quintiles of their respective distributions across all individuals. The mid category (Q3) was chosen as the reference. Models were adjusted for age at biomarker measurement in 5-year age groups, sex, and CCI. Effect modification by age or sex was investigated using likelihood ratio tests analyzing the joint null hypothesis of no multiplicative interaction using three age groups 64–75, 75–84, and 84–99 as well as sex [20]. No effect modification by age or sex was found for the associations between any of the biomarkers and the odds of reaching age 100 (all *p*-values of the likelihood ratio test were >0.05). In a sensitive analysis, we additionally adjusted the logistic regression models for specific morbidities.

In the third step and in order to see if centenarians displayed homogenous biomarker profiles, we conducted cluster analysis using K-median clustering using the Miclust R package (see supplemental materials for further details) [21]. Potential differences in biomarker values between the centenarian clusters and non-centenarians were explored by comparing the respective quantiles of each biomarker distribution among clusters. Note that results from quantile regressions are included in the supplemental materials. Age-stratified analyses (79 years old or less and 80 years old or more) were also conducted as a sensitivity analysis. These results are available in the supplemental materials.

All statistical analyses were conducted using R (version 4.1.2; R Foundation for Statistical Computing, Vienna, Austria).

## Results

Of 44,636 participants, 5851 (13.1%) died before their 80th birthday, 21,234 (47.6%) between their 80th and 90th birthdays, 16,327 (36.6%) between their 90th and 100th birthdays, and 1224 (2.7%) became centenarians. The mean age (SD) at first biomarker measurement was 79.6 (7.5) years for centenarians and 76.7 (6.2) years for non-centenarians. Half of the participants were followed for more than 10 years after biomarker assessment and 13% were followed for more than 20 years. The mean follow-up time was 11.0 (SD 7.4) years. Table 1 shows baseline characteristics for centenarians and non-centenarians. The proportion of females was higher in centenarians (84.6%) than in non-centenarians (61.2%). Despite being on average older at first blood measurement, the prevalence of morbidities was lower among individuals becoming centenarians than among non-centenarians. The proportion of participants with a CCI ≥ 2 was 3.7% in centenarians and 13.8% in non-centenarians. Congestive heart failure was the most frequent morbidity with a prevalence of 2.6% in centenarians compared to 8.7% in non-centenarians.

**Table 1:** Baseline characteristics for centenarians and non-centenarians

	Centenarian (*N*=1224)	Non-centenarian (*N*=43,412)
Age at baseline measurement, *N* (%)^a^		
64–69	125 (10.2%)	6613 (15.2%)
70–74	230 (18.8%)	10,362 (23.9%)
75–79	312 (25.5%)	13,821 (31.8%)
80–84	271 (22.1%)	8299 (19.1%)
85–89	159 (13.0%)	3456 (8.0%)
90–94	94 (7.7%)	790 (1.8%)
95–99	33 (2.7%)	71 (0.2%)
Female, *N* (%)	1035 (84.6%)	26,567 (61.2%)
Comorbidities^a^, *N* (%)		
Myocardial infarction	13 (1.1%)	2259 (5.2%)
Congestive heart failure	32 (2.6%)	3778 (8.7%)
Peripheral vascular disease	5 (0.4%)	701 (1.6%)
Cerebrovascular disease	24 (2.0%)	2650 (6.1%)
Chronic obstructive pulmonary disease	3 (0.2%)	708 (1.6%)
Chronic other pulmonary disease	14 (1.1%)	928 (2.1%)
Rheumatic disease	10 (0.8%)	941 (2.2%)
Dementia	3 (0.2%)	486 (1.1%)
Diabetes without chronic complication	6 (0.5%)	1389 (3.2%)
Peptic ulcer disease	12 (1.0%)	817 (1.9%)
Malignancy	35 (2.9%)	2634 (6.1%)
Charlson Comorbidity Index, *N* (%)		
0	1079 (88.2%)	31,508 (72.6%)
1	100 (8.2%)	5926 (13.7%)
2 or more	45 (3.7%)	5978 (13.8%)

^a^At the time of first blood sample

**Fig. 1:**
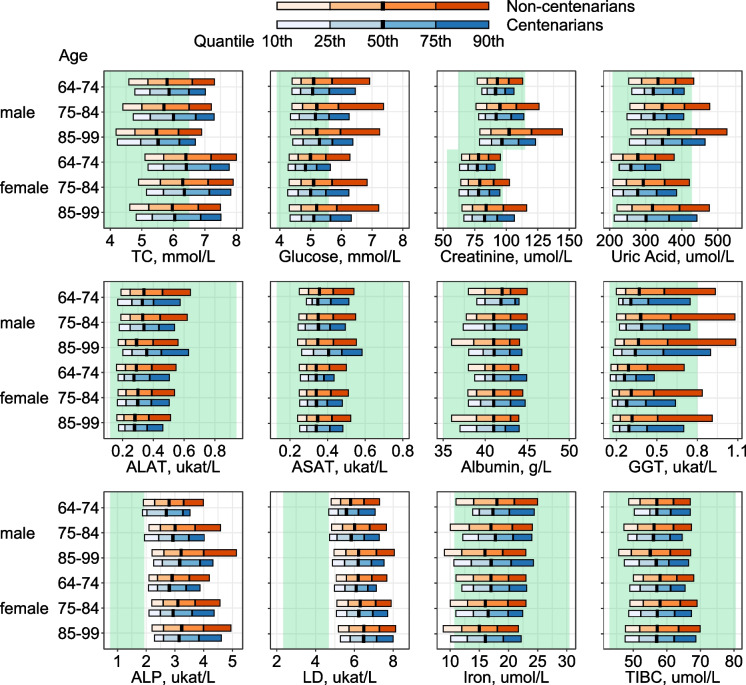
Quantiles (10th, 25th, 50th, 75th, 90th) of biomarkers for centenarians and non-centenarians. Green areas show each biomarker’s normal range based on commonly used clinical thresholds (see supplemental table 1 for further details). Multiple imputed data were used and 44,636 participants were included. TC, total cholesterol; ALAT, alanine aminotransferase; ASAT, aspartate aminotransferase; GGT, gamma-glutamyl transferase; ALP, alkaline phosphatase; TIBC, total iron-binding capacity

Figure 1 shows quantiles of each biomarker distribution by sex and age at measurement for centenarians and non-centenarians. The green area depicts clinically defined normal ranges for each biomarker. For ALAT, ASAT, albumin, iron, and TIBC, both centenarians and non-centenarians fell well within the normal ranges, whereas for ALP and LD both centenarians and non-centenarians had values higher than the normal range. Centenarians overall showed favorable levels of some biomarkers, for example, glucose, creatinine, and uric acid where lower levels are considered healthier. While median values were not different for centenarians and non-centenarians for most biomarkers, differences appeared in the ends of the distributions; however, these differences were not always statistically significant (details on which quantiles were statistically significantly different are found in supplemental table 4). Similar results were observed using complete case data, when additionally stratifying non-centenarians into individuals living up to 90 years, and individuals living 90–99 years, and when showing the mean values and SD (supplemental fig. 1 and 2 and supplemental table 5).

While Fig. 1 primarily allows comparison within each biomarker, supplemental fig. 3 enables a comparison between the biomarkers. The horizontal axis has been standardized using each biomarker’s mean value and standard deviation measured in the entire cohort. Mean biomarker values are shown together with their 95% confidence intervals (CI). The differences in mean values between centenarians and non-centenarians were most pronounced for creatinine and uric acid, however, not statistically significantly different in all age groups. In a sensitivity analysis, we additionally analyzed repeated measures for creatine. These results largely resembled those of the first measurements and centenarians retained creatine levels at second measurement. Moreover, centenarians displayed a smaller change in values between first and second measurements than did non-centenarians (supplemental fig. 4).

**Fig. 2:**
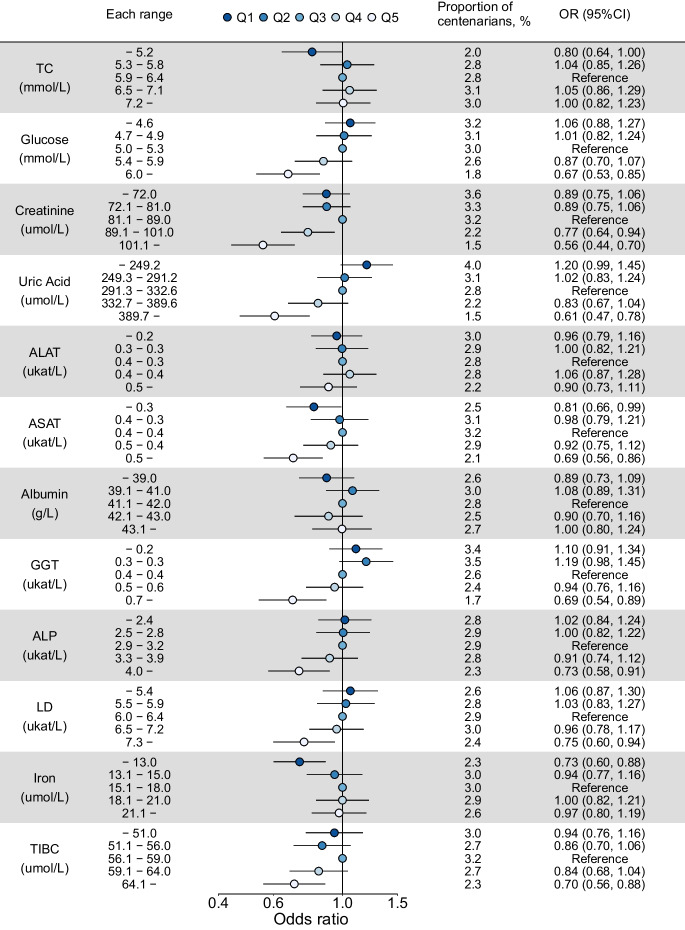
Association between biomarker quintiles and becoming a centenarian estimated with logistic regression adjusted for age, sex, and CCI. Biomarker quintiles are here based on the respective biomarker distribution for all individuals combined (both centenarians and non-centenarians). Multiple imputed data were used and 44,636 participants were included. TC, total cholesterol; ALAT, alanine aminotransferase; ASAT, aspartate aminotransferase; GGT, gamma-glutamyl transferase; ALP, alkaline phosphatase; TIBC, total iron-binding capacity; LD, lactate dehydrogenase; CCI, Charlson Comorbidity Index; OR, odds ratio; CI, confidence interval

Figure 2 shows the proportion of the study population that became centenarians across quintiles of each biomarker. Quintiles are here based on the respective biomarker distribution for all individuals combined. Figure 2 also depicts odds ratios (OR) for becoming a centenarian adjusted for the age at biomarker measurement, sex, and CCI. All but two of the studied biomarkers were associated with the likelihood of reaching age 100. For total cholesterol and iron, higher levels increased the odds, and for glucose, creatinine, uric acid, ASAT, GGT, ALP, LD, and TIBC lower levels increased the odds of becoming a centenarian. A dose-response relationship was found for uric acid; individuals within the lowest quintile had almost twice the chance of reaching age 100 compared to those in the highest quintile. As a comparison, the same analyses using complete case data are found in supplemental fig. 5 showing similar but more pronounced associations. Additionally, adjusting for specific comorbidities did not change the results (supplemental fig. 6). The sensitivity analyses for CRP, ASAT/ALAT ratio, and iron/TIBC ratio showed that low levels of CRP and a high iron/TIBC ratio were associated with a higher chance of becoming a centenarian, while no association was observed between ASAT/ALAT ratio and the chance of reaching age 100 (supplemental fig. 7 and supplemental table 6).

**Fig. 3:**
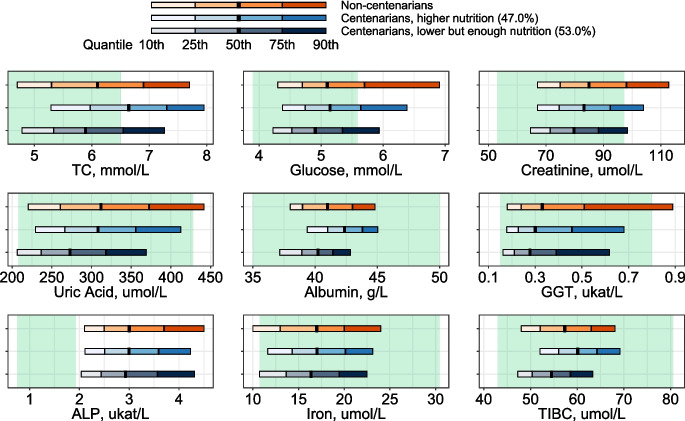
Quantiles (10th, 25th, 50th, 75th, 90th) of biomarkers included in the cluster analysis for centenarian clusters and non-centenarians. Green areas show each biomarker’s normal range based on commonly-used clinical thresholds (see supplemental table 1 for further details). Multiple imputed data were used and 44,636 participants were included. TC, total cholesterol; GGT, gamma-glutamyl transferase; ALP, alkaline phosphatase; TIBC, total iron-binding capacity

Results from the cluster analysis identifying biomarker profiles within the centenarian population are presented in Fig. 3. We identified two subgroups (clusters) encompassing 47.0% and 53.0% of centenarians. We named cluster 1 “higher nutrition” and cluster 2 “lower but enough nutrition” because of a marked difference in TC, albumin, and TIBC and because, albeit lower in cluster 2 than in cluster 1, albumin values were well within the normal range in both clusters. Out of the 12 biomarkers, 9 were identified in the cluster analysis. Cluster 1 displayed higher quantile values than cluster 2 for all included biomarkers but these differences were only statistically significant for TIBC (all quantiles), as well as TC and albumin (see supplemental table 7). Baseline characteristics and a comparison of survival for the two clusters (no difference observed), as well as detailed information on variable selection are provided in the supplemental materials (supplemental table 8, supplemental figs. 8 and 9, and accompanying text). Performing the clustering in age-stratified data and complete case data did not change the results (supplemental figs. 10-12).

## Discussion

Our work is to date the largest study comparing biomarker profiles measured at similar ages earlier in life among exceptionally long-lived individuals and their shorter-lived peers. We compared the biomarker profiles of centenarians to be and their shorter-lived peers, investigated the association between a set of commonly measured biomarkers and the odds of becoming a centenarian, and explored how homogenous the biomarker profiles among the centenarian population were at earlier ages. We found that all included biomarkers except for ALAT and albumin were predictive for the likelihood of reaching age 100. Moreover, more than one decade before their 100th birthday, centenarians had more favorable biomarker levels than their same-aged peers and were rather homogenous in terms of their biomarker profiles. Yet, two distinct profiles were identified. The “higher nutrition” profile resembled more closely the profile of non-centenarians, while “lower but enough nutrition” was characterized by more favorable biomarker levels. However, the differences were small and primarily found for TC, albumin, and TIBC. It is worth noting that the biomarkers accounting for the differences between centenarians and centenarians are not the same as those that distinguish centenarians from each other.

TC, albumin, and TIBC are affected by nutrition and inflammation status as well as by liver function and anemia. They are also used as surrogate markers for nutrition and inflammation status [22]. Yet, we did not observe differences in other biomarkers of inflammation, liver function, and anemia such as uric acid, ASAT, and iron within centenarians. Heterogeneity observed within the centenarian population regarding TC, albumin, and TIBC might thus be related to nutrition rather than inflammation, liver function, and anemia. This could perhaps be related to the research on caloric restriction and its association with longevity [23]. However, the role of caloric restriction for exceptional longevity is not known. Moreover, there was a clear difference in uric acid levels between centenarians and non-centenarians, which might point towards inflammation, rather than—or in addition to—nutrition, playing an important role in determining who reaches age 100.

No association between albumin as a single marker and the likelihood of becoming a centenarian was observed. This contrasts with a previous study in which higher levels of albumin were associated with higher survival chances in older age [8]. However, the previous study analyzed the effect of albumin on continuously measured survival using survival analyses, whereas we examined a dichotomous outcome, i.e., whether or not an individual reached their 100th birthday. It is possible that albumin plays a role in survival at younger ages, but not in the chance of becoming exceptionally old. Moreover, lower albumin levels are associated with weight loss [24] and weight reduction has been reported to start about nine years before death [25]. Since non-centenarians in our study included many people that survived 10 years or more after the biomarkers were measured, the measurement of albumin may have been too far away from the date of death to detect an effect. Our age-stratified analyses indeed indicated a stronger association between albumin and survival for those with near-death measurements, but the differences were not statistically significant.

Our results for the biomarkers of liver and renal function and of inflammation are in line with earlier research. Previous cohort studies have found low levels of creatinine, biomarkers of liver and renal function (cystatin C and cholinesterase), CRP, and inflammation to be predictive for exceptional longevity [7, 8]. Creatinine is a biomarker of renal function and ASAT, GGT, ALP, and LD are biomarkers of liver function. Uric acid can be deemed both a biomarker of inflammation and an indicator of gout [26]. One hypothesis is that alcohol consumption may relate to exceptional longevity, since several alcohol-related biomarkers are higher in non-centenarians compared to centenarians, especially GGT, and ASAT. Uric acid, too, may increase due to alcohol consumption. In sensitivity analyses, we additionally analyzed the ASAT/ALAT ratio which, if above 2, it is a sign of alcoholic liver disease [27] and these analyses showed that the quotient was higher for non-centenarians than centenarians. The difference was, however, rather small. Yet, the relationship between alcohol consumption, the biological response to it, and exceptional longevity may be an interesting topic for future research.

Most individuals, both centenarians and non-centenarians, had values of ALP and LD outside the range considered normal in clinical guidelines. This is likely due to aging and the presence of age-related health conditions [28], as these guidelines are set based on a younger and healthier population. As such, clinically defined normal ranges might not always reflect the optimum for the oldest old. For example, we found that a higher total cholesterol level was associated with a higher chance of becoming centenarian, which stands in contrast to clinical guidelines regarding cholesterol levels [29] but is in line with previous studies showing that high cholesterol is generally favorable for mortality in very old age [30]. A previous cross-sectional study compared cholesterol levels among offspring of exceptionally long-lived individuals and age-matched controls and found slightly higher cholesterol levels among the offspring than controls [9]. Even if they could not observe the life spans of the offspring and controls, it might—in accordance with our work—indicate that high cholesterol levels are more frequently observed among individuals predisposed to survive longer.

Our study has several strengths including a large sample size, a long-term follow-up, representative population (the chance of reaching age 100 was the same in the Amoris cohort as in the general population of Stockholm), and access to high-quality register data allowing a complete follow-up of all participants. Moreover, the biomarkers were analyzed in the same laboratory with a consistently applied and well-documented methodology. However, our study also has limitations that should be considered when interpreting the results. First, we did not have access to all desired biomarkers potentially related to longevity, for example, immunity biomarkers like white blood cells [26]. Immunity has been reported to be crucial in the aging process [17] and, moreover, better information regarding inflammation would have been desirable, although we were able to include uric acid and analyzed sub-samples with information on CRP. Finally, even if biomarkers to some extent reflect lifestyle factors, lifestyle information such as smoking, alcohol consumption, and physical activity would have allowed a better understanding of how lifestyle factors relate to and interact with biomarker levels in exceptionally old age.

In conclusion, already from age 65 onwards, a difference in commonly available biomarkers was observed between individuals who eventually became centenarians and those who did not. *Higher* levels of total cholesterol and iron and *lower* levels of glucose, creatinine, uric acid, ASAT, GGT, ALP, TIBC, and LD were associated with a greater likelihood of becoming a centenarian. While chance likely plays a role for reaching age 100, the differences in biomarker values more than one decade prior death suggest that genetic and/or lifestyle factors, reflected in these biomarker levels may also play a role for exceptional longevity. Our work—to date the largest study on this topic—also shows that centenarians had homogeneous biomarker profiles which underscores the importance of specific biomarker characteristics in research on exceptional longevity.

### Supplementary information

ESM 1 (PDF 342 kb)

## Data Availability

Individual level data used for this study cannot be shared because of the General Data Protection Regulation in Sweden. Access to the data is possible for external researchers after ethical vetting and the establishment of a collaboration agreement. More information on data requests at the AMORIS homepage (https://ki.se/en/imm/amoris).

## References

[1] United nations, department of economic and social affairs, population division. World population prospects 2022, Online Edition. 2022. https://population.un.org/wpp/. Accessed 21 June 2023.

[2] Willcox DC, Willcox BJ, Poon LW (2010). Centenarian studies: important contributors to our understanding of the aging process and longevity. Curr Gerontol Geriatr Res.

[3] Chung WH, Dao RL, Chen LK (2010). The role of genetic variants in human longevity. Ageing Res Rev.

[4] Vetrano DL, Grande G, Marengoni A (2021). Health trajectories in Swedish centenarians. J Gerontol A Biol Sci Med Sci.

[5] Engberg H, Oksuzyan A, Jeune B (2009). Centenarians--a useful model for healthy aging? A 29-year follow-up of hospitalizations among 40,000 Danes born in 1905. Aging Cell.

[6] Ismail K, Nussbaum L, Sebastiani P (2016). Compression of morbidity is observed across cohorts with exceptional longevity. J Am Geriatr Soc.

[7] Arai Y, Martin-Ruiz CM, Takayama M (2015). Inflammation, but not telomere length, predicts successful ageing at extreme old age: a longitudinal study of semi-supercentenarians. EBioMedicine..

[8] Hirata T, Arai Y, Yuasa S (2020). Associations of cardiovascular biomarkers and plasma albumin with exceptional survival to the highest ages. Nat Commun.

[9] Barzilai N, Atzmon G, Schechter C (2003). Unique lipoprotein phenotype and genotype associated with exceptional longevity. JAMA.

[10] Paolisso G, Gambardella A, Ammendola S (1996). Glucose tolerance and insulin action in healthy centenarians. Am J Physiol.

[11] Motta M, Bennati E, Ferlito L (2005). Successful aging in centenarians: myths and reality. Arch Gerontol Geriatr.

[12] Alvarez JA, Medford A, Strozza C (2021). Stratification in health and survival after age 100: evidence from Danish centenarians. BMC Geriatr.

[13] Gellert P, von Berenberg P, Zahn T (2019). Multimorbidity profiles in German centenarians: a latent class analysis of health insurance data. J Aging Health.

[14] Walldius G, Malmstrom H, Jungner I (2017). Cohort profile: the AMORIS cohort. Int J Epidemiol.

[15] Van Hemelrijck M, Harari D, Garmo H (2012). Biomarker-based score to predict mortality in persons aged 50 years and older: a new approach in the Swedish AMORIS study. Int J Mol Epidemiol Genet.

[16] Ludvigsson JF, Appelros P, Askling J (2021). Adaptation of the Charlson Comorbidity Index for register-based research in Sweden. Clin Epidemiol.

[17] Ahadi S, Zhou W, Schussler-Fiorenza Rose SM (2020). Personal aging markers and ageotypes revealed by deep longitudinal profiling. Nat Med.

[18] Denny SD, Kuchibhatla MN, Cohen HJ (2006). Impact of anemia on mortality, cognition, and function in community-dwelling elderly. Am J Med.

[19] Wennberg AM, Ding M, Ebeling M (2021). Blood-based biomarkers and long-term risk of frailty-experience from the Swedish AMORIS cohort. J Gerontol A Biol Sci Med Sci.

[20] Van Buuren S (2018). Flexible imputation of missing data.

[21] Basagana X, Barrera-Gomez J, Benet M (2013). A framework for multiple imputation in cluster analysis. Am J Epidemiol.

[22] Stromberg BV, Davis RJ, Danziger LH (1982). Relationship of serum transferrin to total iron binding capacity for nutritional assessment. JPEN J Parenter Enteral Nutr.

[23] Dorling JL, Martin CK, Redman LM (2020). Calorie restriction for enhanced longevity: the role of novel dietary strategies in the present obesogenic environment. Ageing Res Rev.

[24] Bello AK, de Zeeuw D, El Nahas M (2007). Impact of weight change on albuminuria in the general population. Nephrol Dial Transplant.

[25] Alley DE, Metter EJ, Griswold ME (2010). Changes in weight at the end of life: characterizing weight loss by time to death in a cohort study of older men. Am J Epidemiol.

[26] Ruggiero C, Cherubini A, Ble A (2006). Uric acid and inflammatory markers. Eur Heart J.

[27] Sorbi D, Boynton J, Lindor KD (1999). The ratio of aspartate aminotransferase to alanine aminotransferase: potential value in differentiating nonalcoholic steatohepatitis from alcoholic liver disease. Am J Gastroenterol.

[28] Shimizu Y, Ichihara K, Asia-Pacific Federation of Clinical B (2015). Sources of variation analysis and derivation of reference intervals for ALP, LDH, and amylase isozymes using sera from the Asian multicenter study on reference values. Clin Chim Acta.

[29] Mach F, Baigent C, Catapano AL (2020). 2019 ESC/EAS Guidelines for the management of dyslipidaemias: lipid modification to reduce cardiovascular risk. Eur Heart J.

[30] Ding M, Wennberg A, Ek S (2021). The association of apolipoproteins with later-life all-cause and cardiovascular mortality: a population-based study stratified by age. Sci Rep.

