# Correction to: Blood biomarker profiles and exceptional longevity: comparison of centenarians and non‑centenarians in a 35‑year follow‑up of the Swedish AMORIS cohort

**DOI:** 10.1007/s11357-023-00996-y

**Published:** 2023-11-04

**Authors:** Shunsuke Murata, Marcus Ebeling, Anna C. Meyer, Katharina Schmidt‑Mende, Niklas Hammar, Karin Modig

**Affiliations:** 1 Unit of epidemiology, Institute of Environmental Medicine, Karolinska Institutet, Box 210, 17177 Stockholm, Sweden; 2 Department of Preventive Medicine and Epidemiology, National Cerebral and Cardiovascular Center, Suita, Japan; 3 Laboratory of Population Health, Max Planck Institute for Demographic Research, Rostock, Germany; 4 Academic Primary Health Care Centre, Stockholm Region, Stockholm, Sweden; 5 Division of Family Medicine and Primary Care, Department of Neurobiology, Care Sciences and Society, Karolinska Institutet, Huddinge, Sweden

**Correction to: GeroScience**

**https://doi.org/10.1007/s11357-023-00936-w**

In our manuscript, part of the interpretation of Figure 2 is based on an earlier version of the Figure that had the lowest quintile as the reference. During the review process, the reference point was changed to the middle category. This changed some of the interpretations, for example, the statement “Higher levels of total cholesterol was associated with increased odds of reaching 100 years” should rather be “low cholesterol was associated with a reduced likelihood of reaching the age of 100”. Having high cholesterol neither increases nor decreases the probability of living to 100 years of age. The same is true also for some other biomarkers and the interpretation of Figure 2 should be: Belonging to the lowest quintiles of total cholesterol and iron or the highest quintiles of glucose, uric acid, gamma-glutamyl transferase, alkaline phosphatase, lactate dehydrogenase, and total iron-binding capacity was associated with a lower likelihood of reaching 100 years. For creatinine, both the highest and second highest quintiles were associated with a lower chance of reaching age 100, and for aspartate aminotransferase the highest and lowest quintiles were associated with a lower likelihood of reaching 100 years.

