# Supplementary material for: Blood biomarker profiles and exceptional longevity: comparison of centenarians and non-centenarians in a 35-year follow-up of the Swedish AMORIS cohort
Source: GeroScience. 2023 Sep 19;46(2):1693–702. doi: 10.1007/s11357-023-00936-w (PMC10828184; doi:10.1007/s11357-023-00936-w)
Supplement: Supplementary file 1 — (PDF 342 kb) [file 11357_2023_936_MOESM1_ESM.pdf]

Contents

Supplemental table 1: Type and normal range of each biomarker.

Supplemental table 2: Mean (standard deviation) of biomarkers between centenarians and non-centenarians using complete case samples and imputed samples and proportion of biomarkers missing.

Supplemental table 3: Baseline characteristics between older adults with and without at least one missing value.

Additional methods of statistical analysis

Supplemental table 4: Quantile regression comparing differences in 25th, 50th (median), 75th quantiles between centenarians and non-centenarians.

Supplemental figure 1: Biomarker quantiles (10th, 25th, 50th, 75th, 90th) of biomarkers for centenarians and non-centenarians using complete case data.

Supplemental figure 2: Biomarker quantiles (10th, 25th, 50th, 75th, 90th) among centenarians, nonagenarians, and sexagenarians, septuagenarians, and octogenarians.

Supplemental table 5: Mean (standard deviation) of biomarkers between centenarians and non-centenarians with stratification by age and sex.

Supplemental figure 3: Biomarkers' means and 95% confidence intervals standardized using mean and standard deviation observed in the total study population for centenarians and non-centenarians.

Supplemental figure 4: Quantiles (10th, 25th, 50th, 75th, 90th) and means (standard deviation) of first and second creatinine measurements, and their changes between centenarians and non-centenarians.

Supplemental figure 5: Association between biomarker quintiles and the chance of becoming a centenarian: logistic regression using complete case data.

Supplemental figure 6: Association between biomarker quintiles and becoming a centenarian estimated with logistic regression adjusted for age, sex, and each comorbidities using multiply imputed data.

Supplemental figure 7: Quantiles (10th, 25th, 50th, 75th, 90th) of CRP, ASAT/ALAT, and iron/TIBC between centenarians and non-centenarians.

Supplemental table 6: Association of CRP, ASAT/ALAT, and iron/TIBC quintile with the chance of becoming a centenarian: logistic regression.

Supplemental table 7: Quantile regression comparing differences in 25th, 50th (median), 75th quantiles between clusters.

Detailed results of the clustering process and variable selection

Supplemental figure 8: Detailed results of the K-medians clustering procedure using multiply imputed data.

Supplemental table 8: Characteristics of individuals in each cluster.

Supplemental figure 9: Comparison of suvival probability between clusters: Kaplan-Meier survival curve and Cox proprotional hazard model.

Supplemental figure 10: Quantiles (10th, 25th, 50th, 75th, 90th) of selected biomarkers among each centenarian cluster and non-centenarians restricting to participants whose blood was sampled before age 80 years.

Supplemental figure 11: Quantiles (10th, 25th, 50th, 75th, 90th) of selected biomarkers among each centenarian cluster and non-centenarians restricting to participants whose blood was sampled at age 80 or older.

Supplemental figure 12: Quantiles (10th, 25th, 50th, 75th, 90th) of selected biomarkers among each centenarian cluster and non-centenarians using complete case data.

Supplemental table 1: Type and normal range of each biomarker.

Biomarkers	Type	Normal range	Reference
Total cholesterol (mmol/L)	Metabolic functioning	<6.5	[1]
Glucose (mmol/L)	Metabolic functioning	3.9 – 5.59	[2]
Creatinine (umol/L)	Kidney functioning	61.9 – 114.9 for men<br>53 – 97.2 for women	[3]
Uric acid (umol/L)	Inflammations	208 – 428	[4]
ALAT (ukat/L)	Liver functioning	0.12 – 0.94	[5]
ASAT (ukat/L)	Liver functioning	0.13 – 0.8	[5]
Albumin (g/L)	Liver functioning and nutrition	35 – 50	[5]
GGT (ukat/L)	Liver functioning	0.15 – 0.8	[5]
Alkaline phosphatase (ukat/L)	Liver functioning	0.75 – 1.93	[5]
Lactate dehydrogenase (ukat/L)	Liver functioning	2.34 – 4.68	[6]
Iron (umol/L)	Anemia	10.74 – 30.43	[7]
TIBC (umol/L)	Anemia	42.96 – 80.55	[8]

ALAT, alanine aminotransferase; ASAT, aspartate aminotransferase; GGT, Gamma-glutamyl transferase; TIBC, total iron-binding capacity

Supplemental table 2: Mean (standard deviation) of biomarkers between centenarians and non-centenarians using complete case samples and imputed samples and proportion of biomarkers missing.

Biomarkers	Missing, N (%)	Complete case sample, Mean (SD)		Imputed sample, Mean (SD)	
		Centenarians	Non-centenarians	Centenarians	Non-centenarians
TC (mmol/L)	12,598 (28.2%)	6.3 (1.1)	6.2 (1.2)	6.3 (1.1)	6.1 (1.2)
Glucose (mmol/L)	14,030 (31.4%)	5.1 (1.1)	5.5 (1.7)	5.2 (1.3)	5.5 (1.8)
Creatinine (umol/L)	3,136 (7.0%)	81.5 (14.0)	88.0 (23.3)	82.5 (15.7)	89.1 (26.1)
Uric acid (umol/L)	12,239 (27.4%)	286.8 (70.6)	315.5 (83.4)	297.2 (79.8)	323.9 (91.4)
ALAT (ukat/L)	9,045 (20.3%)	0.3 (0.6)	0.4 (0.3)	0.3 (0.5)	0.4 (0.4)
ASAT (ukat/L)	9,535 (21.4%)	0.4 (0.4)	0.4 (0.3)	0.4 (0.3)	0.4 (0.3)
Albumin (g/L)	8,712 (19.5%)	41.0 (2.5)	41.0 (2.7)	41.1 (2.7)	41.1 (2.9)
GGT (ukat/L)	10,790 (24.2%)	0.4 (0.4)	0.5 (0.8)	0.4 (0.5)	0.5 (1.0)
ALP (ukat/L)	10,371 (23.2%)	3.0 (1.3)	3.2 (1.7)	3.2 (1.4)	3.3 (1.9)
LD (ukat/L)	15,413 (34.5%)	6.2 (1.7)	6.3 (1.8)	6.3 (1.6)	6.4 (1.7)
Iron (umol/L)	12,780 (28.6%)	17.1 (4.7)	17.1 (5.4)	16.9 (4.9)	16.9 (5.5)
TIBC (umol/L)	13,571 (30.4%)	57.2 (7.2)	58.0 (7.8)	57.5 (7.5)	58.0 (8.0)

SD, standard deviation; TC, total cholesterol; ALAT, alanine aminotransferase; ASAT, aspartate aminotransferase; GGT, gamma-glutamyl transferase; ALP, alkaline phosphatase; LD, lactate dehydrogenase; TIBC, total iron-binding capacity

Supplemental table 3: Baseline characteristics between older adults with and without at least one missing value.

	with missing (N=17,970)	without missing (N=26,666)
Age at baseline measurement, N (%) *		
64-69	428 (2.4%)	6,310 (23.7%)
70-74	2,518 (14.0%)	8,074 (30.3%)
75-79	7,077 (39.4%)	7,056 (26.5%)
80-84	4,810 (26.8%)	3,760 (14.1%)
85-89	2,384 (13.3%)	1,231 (4.6%)
90-94	660 (3.7%)	224 (0.8%)
95-99	93 (0.5%)	11 (0.0%)
Female, N (%)	11,150 (62.0%)	16,452 (61.7%)
Comorbidities*, N (%)		
Myocardial infarction	1,236 (6.9%)	1,036 (3.9%)
Congestive heart failure	2,223 (12.4%)	1,587 (6.0%)
Peripheral vascular disease	436 (2.4%)	270 (1.0%)
Cerebrovascular disease	1,663 (9.3%)	1,011 (3.8%)
Chronic obstructive pulmonary disease	421 (2.3%)	290 (1.1%)
Chronic other pulmonary disease	521 (2.9%)	421 (1.6%)
Rheumatic disease	492 (2.7%)	459 (1.7%)
Dementia	445 (2.5%)	44 (0.2%)
Diabetes without chronic complication	870 (4.8%)	525 (2.0%)
Peptic ulcer disease	467 (2.6%)	362 (1.4%)
Malignancy	1,394 (7.8%)	1,275 (4.8%)
Charlson comorbidity index, N (%)		
0	11,400 (63.4%)	21,187 (79.5%)
1	3,129 (17.4%)	2,897 (10.9%)
2 or more	3,441 (19.1%)	2,582 (9.7%)

*At the time of first blood sample.

Additional methods of statistical analysis

In multiple imputation process, we created 100 imputed data sheets assuming missing at random. To avoid model misspecification, random forests were used to predict missing values [9]. The predictors in the random forest were all available biomarker values, age, sex, and specific comorbidities. The imputation was conducted using the mice and randomForest packages in R [10, 11]. Rubin's rule was used to combine the results of the 100 individual imputations.
In the first step investigating differences in the biomarkers' destributions, quantile regression were built to compare 75th quantile of biomarkers with adjustment age and sex. We compared the distribution of biomarker values using the 10th quantile, 25th quantile, median (50th quantile), 75th quantile, and 90th quantile among three groups: people who died before their 90th birthday (sexagenarians, septuagenarians, and octogenarians), people who died between their 90th birthday and 100th birthday (nonagenarians), and people reaching their 100th birthday (centenarians). Those quantiles were also compared with the normal range of each biomarker based on previously established evidence (Supplemental table 1). In a subsample with a repeated creatinine measurement within 5 years of the first measurement, we further compared quantiles and means (standard deviation) of first and second measuremed creatinine as well as the mean (standard deviation) change in supplemental figure 4. As a sensitivity analysis, we also analyzed CRP (an inflammatory marker) which was measured for a subset of participants, the iron/TIBC ratio as an indicator of iron deficiency, and the ASAT/ALAT ratio as a marker of liver status in the supplemental figure 7 and table 6 [12, 13]. We compared mean values and 95% confidence intervals (CI) between centenarians and non-centenarians using ordinary least squares with estimated marginal means [14].
In the third step investigating the variation in biomarker profiles, we used K-medians clustering since we observed some outliers in the biomarker distributions. This study followed the steps of clustering analyses using multiply imputed data sets presented previously [15]. We used forward sequential selection to reduce the variables used in the clustering process [16]. The optimal number of clusters was explored and chosen based on the most frequently selected number of clusters in 100 imputed data sets using CritCF [15]. We included all biomarkers that appeared at least once in any K-medians variable selection of 100 multiply imputed data sets due to sufficient sample size in our data [15]. Some participants can be allocated to different clusters when different imputed data sets are used. The algorithm to determine cluster membership in our final model was based on all scenarios estimated with 100 imputed data sets. After the clustering procedure, baseline characteristics were compared between clusters and survival differences were also examined between clusters using Kaplan-Meier curves and Cox proportional hazards model.

Supplemental table 4: Quantile regression comparing differences in 25th, 50th (median), 75th quantiles between centenarians and non-centenarians.

biomarker	age	Male			Female		
		p of 25th	*p* of 50th	*p* of 75th	*p* of 25th	*p* of 50th	*p* of 75th
TC	64-74	0.466	0.603	1.000	1.000	1.000	0.222
	75-84	0.689	0.068	0.023*	0.020*	1.000	0.372
	85-99	0.081	0.414	0.354	0.032*	0.017*	0.179
Glucose	64-74	1.000	1.000	0.418	0.064	0.003*	<0.0001*
	75-84	0.376	0.088	0.007*	1.000	0.004*	0.008*
	85-99	0.784	0.411	0.684	0.320	0.361	0.028*
Creatinine	64-74	1.000	0.574	0.109	0.227	0.068	0.024*
	75-84	0.047*	0.182	0.307	1.000	0.371	0.039*
	85-99	0.897	0.159	0.114	0.583	0.074	0.018*
Uric Acid	64-74	0.558	0.176	0.732	0.009*	<0.0001*	<0.0001*
	75-84	0.331	0.065	<0.0001*	0.061	<0.001*	0.012*
	85-99	0.811	0.415	0.134	0.238	0.019*	0.001*
ALAT	64-74	0.656	1.000	0.183	0.207	0.041*	0.242
	75-84	0.248	0.711	0.670	1.000	1.000	1.000
	85-99	0.244	0.432	0.238	0.390	1.000	0.519
ASAT	64-74	0.016*	0.226	0.652	0.226	1.000	<0.001*
	75-84	0.537	0.396	0.571	1.000	0.170	0.045*
	85-99	0.074	0.033*	0.174	0.443	0.352	0.620
Albumin	64-74	1.000	1.000	0.044*	1.000	1.000	1.000
	75-84	1.000	1.000	1.000	1.000	1.000	0.045*
	85-99	0.135	1.000	1.000	1.000	0.068	1.000
GGT	64-74	0.209	0.003*	0.059	0.007*	<0.001*	<0.0001*
	75-84	1.000	0.313	0.257	0.004*	<0.0001*	<0.001*
	85-99	0.843	0.847	0.853	0.485	0.624	0.035*
ALP	64-74	0.031*	1.000	1.000	1.000	0.153	0.040*
	75-84	1.000	1.000	0.277	0.167	0.065	0.314
	85-99	0.695	0.728	0.707	1.000	1.000	0.310
LD	64-74	0.545	0.113	0.357	0.430	0.124	0.012*
	75-84	0.074	0.284	0.147	0.258	0.080	0.333
	85-99	0.482	1.000	0.037*	1.000	1.000	0.335
Iron	64-74	0.191	0.205	0.416	1.000	1.000	0.171
	75-84	0.127	0.271	1.000	0.020*	1.000	0.172
	85-99	0.187	0.189	0.528	0.135	0.147	0.113
TIBC	64-74	0.045*	1.000	0.591	0.244	0.069	<0.001*
	75-84	0.364	1.000	1.000	0.105	0.054	0.010*
	85-99	0.336	0.360	0.607	1.000	0.305	0.284

TC, total cholesterol; ALAT, alanine aminotransferase; ASAT, aspartate aminotransferase; GGT, gamma-glutamyl transferase; ALP, alkaline phosphatase; LD, lactate dehydrogenase; TIBC, total iron-binding capacity. Complete case data included 707 centenarians and 25,959 non-centenarians. *$p<0.05$.

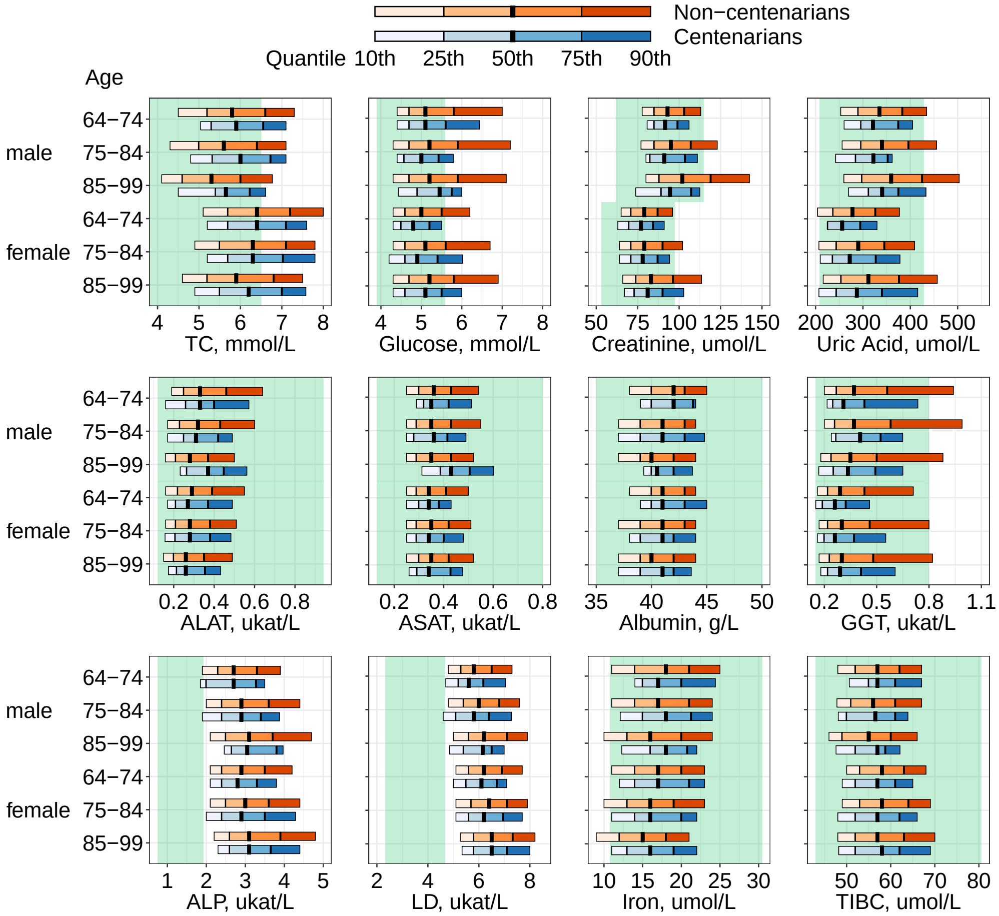

Supplemental figure 1: Biomarker quantiles (10th, 25th, 50th, 75th, 90th) of biomarkers for centenarians and non-centenarians using complete case data.

Green areas show each biomarker's normal range based on commonly-used clinical thresholds (see Supplemental table 1 for further details). Complete case data were used and 26,666 participants were include.

TC, total cholesterol; ALAT, alanine aminotransferase; ASAT, aspartate aminotransferase; GGT, gamma-glutamyl transferase; ALP, alkaline phosphatase; TIBC, total iron-binding capacity.

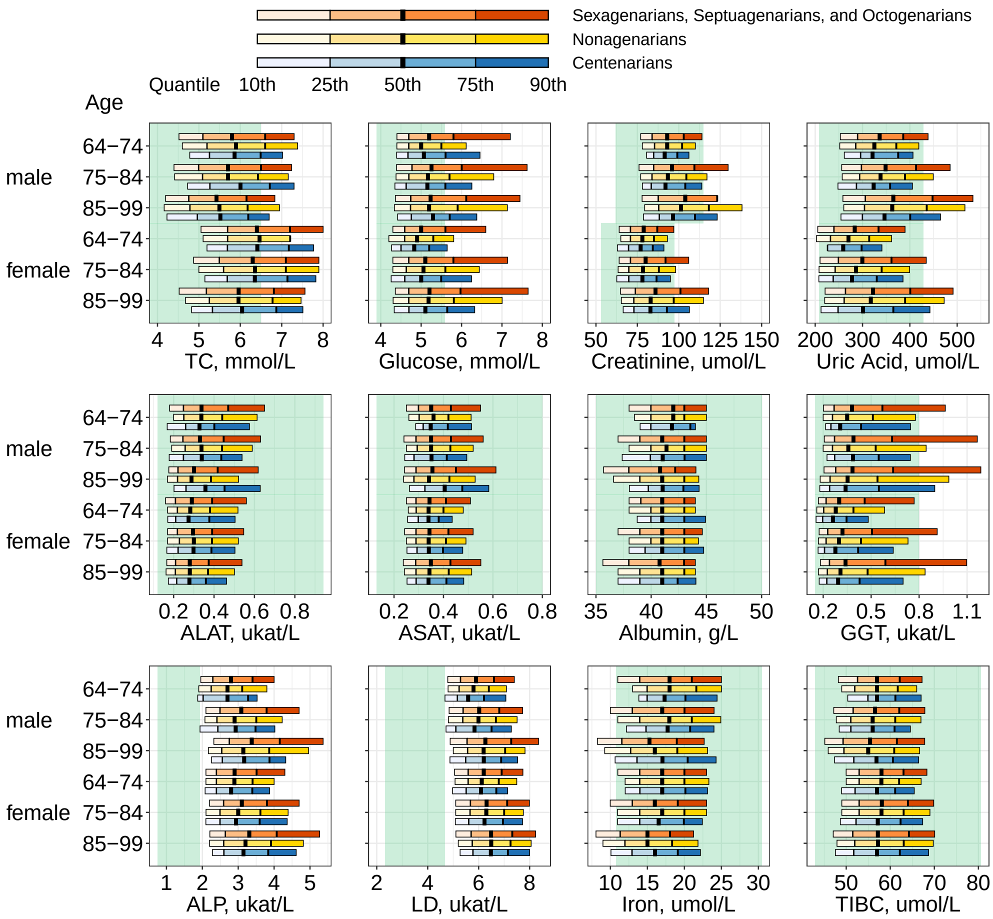

Supplemental figure 2: Biomarker quantiles (10th, 25th, 50th, 75th, 90th) among centenarians, nonagenarians, and sexagenarians, septuagenarians, and octogenarians.

Green areas show each biomarker's normal range. Sexagenarians, septuagenarians, and octogenarians include people who died before their 90th birthday, nonagenarians include people who died between their 90th and 100th birthday, and centenarians include people reaching their 100th birthday. Multiply imputed data were used and 44,636 participants were included.

TC, total cholesterol; ALAT, alanine aminotransferase; ASAT, aspartate aminotransferase; GGT, gamma-glutamyl transferase; ALP, alkaline phosphatase; LD, lactate dehydrogenase; TIBC, total iron-binding capacity

Supplemental table 5: Mean (standard deviation) of biomarkers between centenarians and non-centenarians with stratification by age and sex.

Blood	Sex	64 - 74 years old		75 - 84 years old		85 - 99 years old	
		centenarians	non-centenarians	centenarians	non-centenarians	centenarians	non-centenarians
Total cholesterol (mmol/L)	Male	5.9 (1.0)	5.9 (1.1)	6.0 (1.0)	5.8 (1.1)	5.5 (1.0)	5.5 (1.1)
	Female	6.5 (1.1)	6.5 (1.2)	6.5 (1.1)	6.4 (1.2)	6.1 (1.1)	6.0 (1.2)
Glucose (mmol/L)	Male	5.2 (0.9)	5.6 (1.9)	5.3 (1.2)	5.7 (1.9)	5.4 (1.5)	5.7 (2.0)
	Female	4.9 (0.8)	5.3 (1.5)	5.3 (1.4)	5.5 (1.8)	5.3 (1.3)	5.6 (2.1)
Creatinine (umol/L)	Male	92.6 (10.5)	95.5 (24.2)	94.6 (16.2)	100.0 (30.9)	100.9 (21.8)	110.1 (40.0)
	Female	77.2 (10.8)	79.8 (14.9)	78.7 (12.9)	82.5 (21.6)	85.0 (17.5)	88.9 (26.0)
Uric acid (umol/L)	Male	332.7 (56.5)	339.5 (73.2)	326.2 (63.9)	358.2 (92.5)	355.8 (85.8)	380.5 (108.9)
	Female	265.0 (58.7)	286.3 (72.2)	288.4 (73.4)	306.8 (88.2)	317.9 (99.0)	337.0 (105.9)
ALAT (ukat/L)	Male	0.4 (0.2)	0.4 (0.3)	0.4 (0.2)	0.4 (0.4)	0.4 (0.2)	0.4 (0.6)
	Female	0.3 (0.2)	0.3 (0.3)	0.4 (0.7)	0.4 (0.3)	0.3 (0.3)	0.3 (0.3)
ASAT (ukat/L)	Male	0.4 (0.1)	0.4 (0.2)	0.4 (0.1)	0.4 (0.3)	0.4 (0.2)	0.4 (0.6)
	Female	0.3 (0.1)	0.4 (0.2)	0.4 (0.5)	0.4 (0.2)	0.4 (0.2)	0.4 (0.3)
Albumin (g/L)	Male	41.7 (2.3)	41.5 (2.6)	41.3 (3.0)	41.2 (3.0)	41.1 (2.6)	40.4 (3.5)
	Female	41.4 (2.4)	41.2 (2.5)	41.2 (2.6)	41.1 (2.9)	40.7 (3.1)	40.5 (3.3)
GGT (ukat/L)	Male	0.4 (0.4)	0.5 (0.9)	0.5 (0.5)	0.6 (1.3)	0.5 (0.8)	0.7 (1.6)
	Female	0.3 (0.3)	0.4 (0.6)	0.4 (0.5)	0.5 (0.9)	0.4 (0.6)	0.5 (1.1)
Alkaline phosphatase (ukat/L)	Male	2.7 (0.8)	3.0 (1.6)	3.0 (1.0)	3.4 (2.2)	3.4 (2.1)	3.8 (3.3)
	Female	2.9 (0.8)	3.1 (1.2)	3.1 (1.2)	3.3 (1.7)	3.5 (2.0)	3.6 (2.1)
Lactate dehydrogenase (ukat/L)	Male	5.7 (1.1)	6.0 (1.9)	5.9 (1.3)	6.2 (1.7)	6.3 (1.3)	6.5 (1.7)
	Female	6.1 (0.9)	6.3 (1.6)	6.4 (1.9)	6.5 (1.6)	6.6 (1.3)	6.6 (1.9)
Iron (umol/L)	Male	18.1 (4.6)	18.2 (5.7)	17.8 (5.0)	17.2 (5.8)	17.2 (5.9)	16.0 (5.7)
	Female	17.3 (4.5)	17.1 (5.1)	16.8 (4.8)	16.5 (5.2)	16.1 (4.9)	15.3 (5.4)
TIBC (umol/L)	Male	58.0 (6.0)	57.7 (7.4)	56.4 (7.2)	57.1 (8.1)	56.8 (7.9)	55.7 (8.6)
	Female	57.3 (7.0)	58.5 (7.4)	57.8 (7.6)	58.7 (8.1)	57.5 (8.2)	58.0 (8.9)

Multiply imputed data were used and 44,636 participants were included.

ALAT, alanine aminotransferase; ASAT, aspartate aminotransferase; GGT, gamma-glutamyl transferase; TIBC, total iron-binding capacity

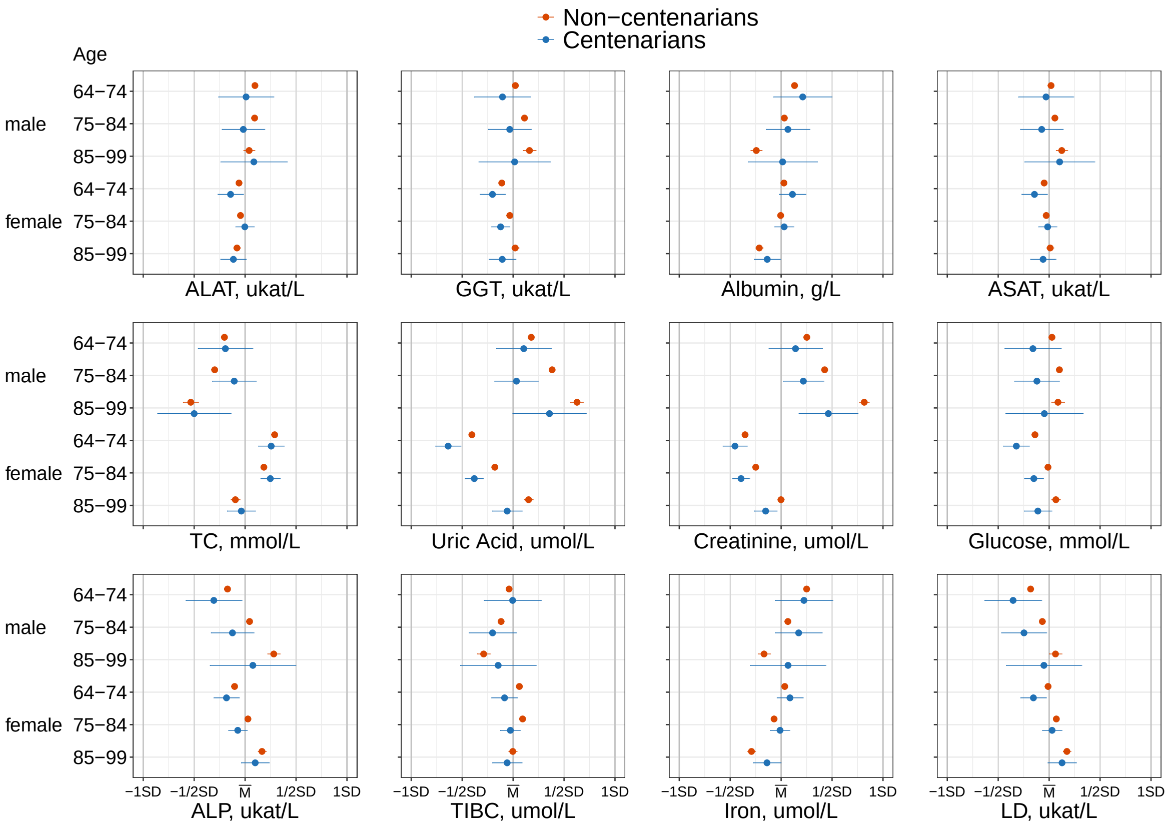

Supplemental figure 3: Biomarkers' means and 95% confidence intervals standardized using mean and standard deviation observed in the total study population for centenarians and non-centenarians.

Multiply imputed data were used and 44,636 participants were included.

TC, total cholesterol; ALAT, alanine aminotransferase; ASAT, aspartate aminotransferase; GGT, gamma-glutamyl transferase; ALP, alkaline phosphatase; TIBC, total iron-binding capacity; $\overline{\mathrm{M}}$, mean in total study population; SD, standard deviation in total study population

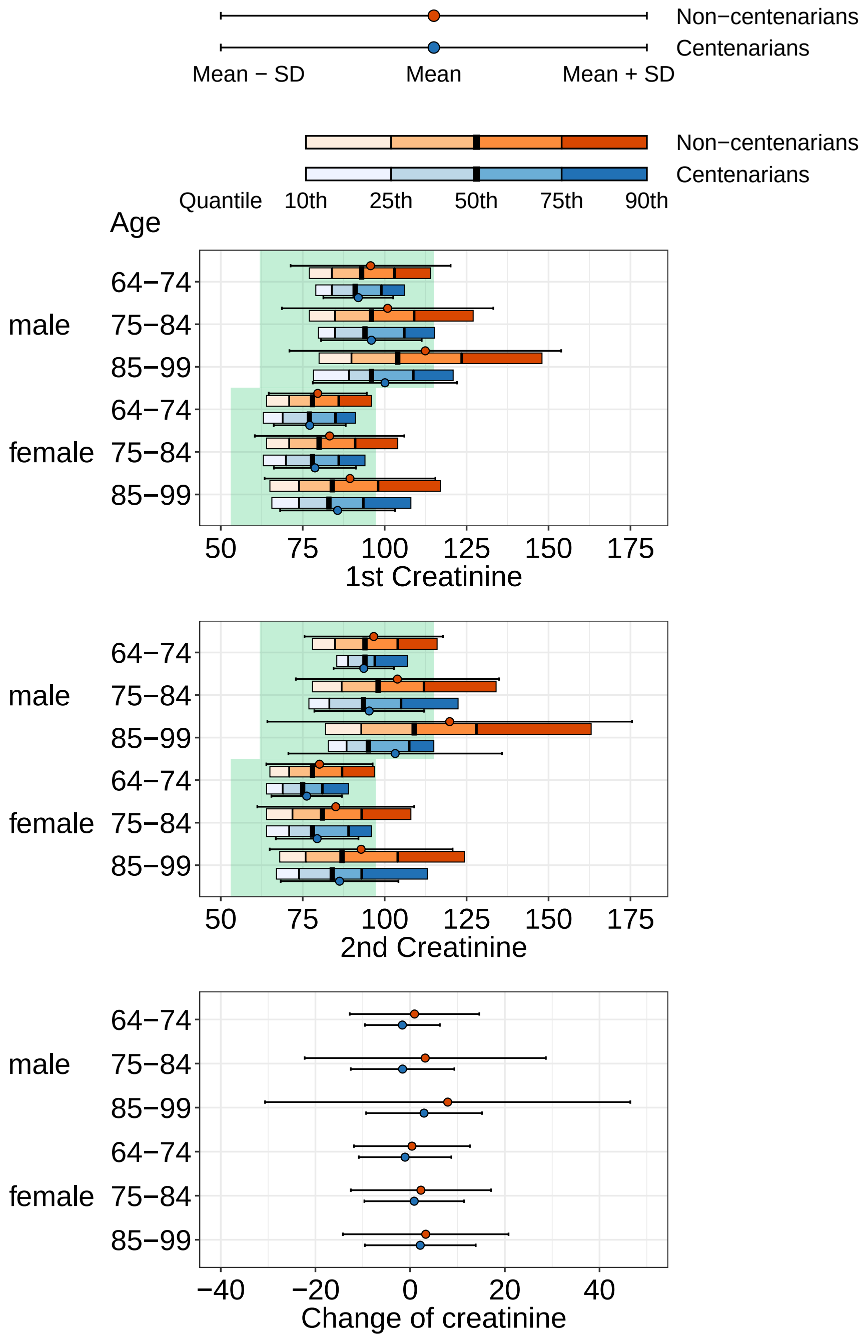

Supplemental figure 4: Quantiles (10th, 25th, 50th, 75th, 90th) and means (standard deviation) of first and second creatinine measurements, and their changes between centenarians and non-centenarians.
SD, standard deviation. Creatinine was measured twice in 51.6% (N=21,433) of participants, which were included in the analysis.

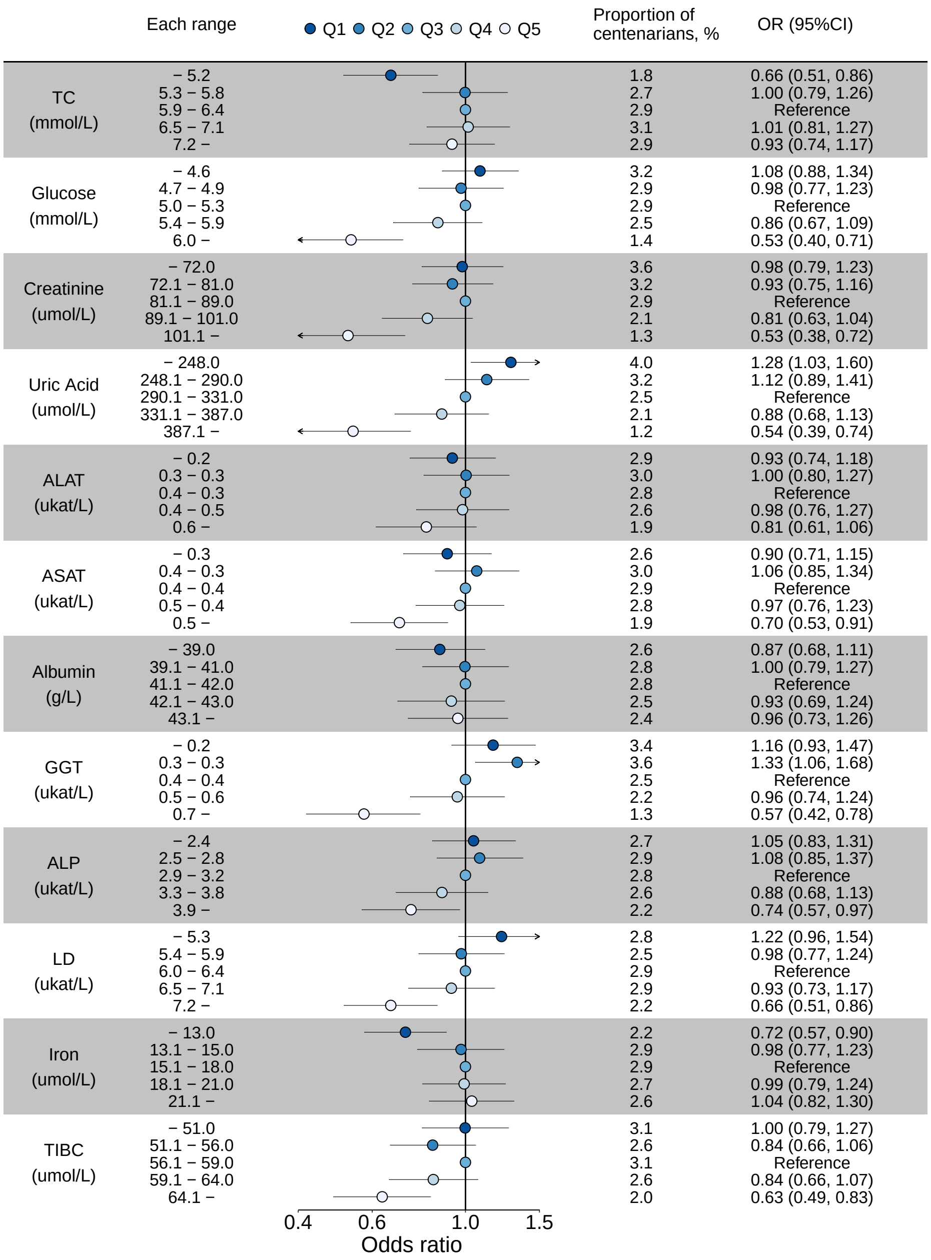

Supplemental figure 5: Association between biomarker quintiles and the chance of becoming a centenarian: logistic regression using complete case data.

Each model was adjusted by age, sex, and CCI. Complete case data included 26,666 participants.

TC, total cholesterol; ALAT, alanine aminotrans,ferase; ASAT, aspartate aminotransferase; GGT, gamma-glutamyl transferase; ALP, alkaline phosphatase; LD, lactate dehydrogenase; TIBC, total iron-binding capacity; CCI, Charlson comorbidity index; OR, odds ratio; CI, confidence interval

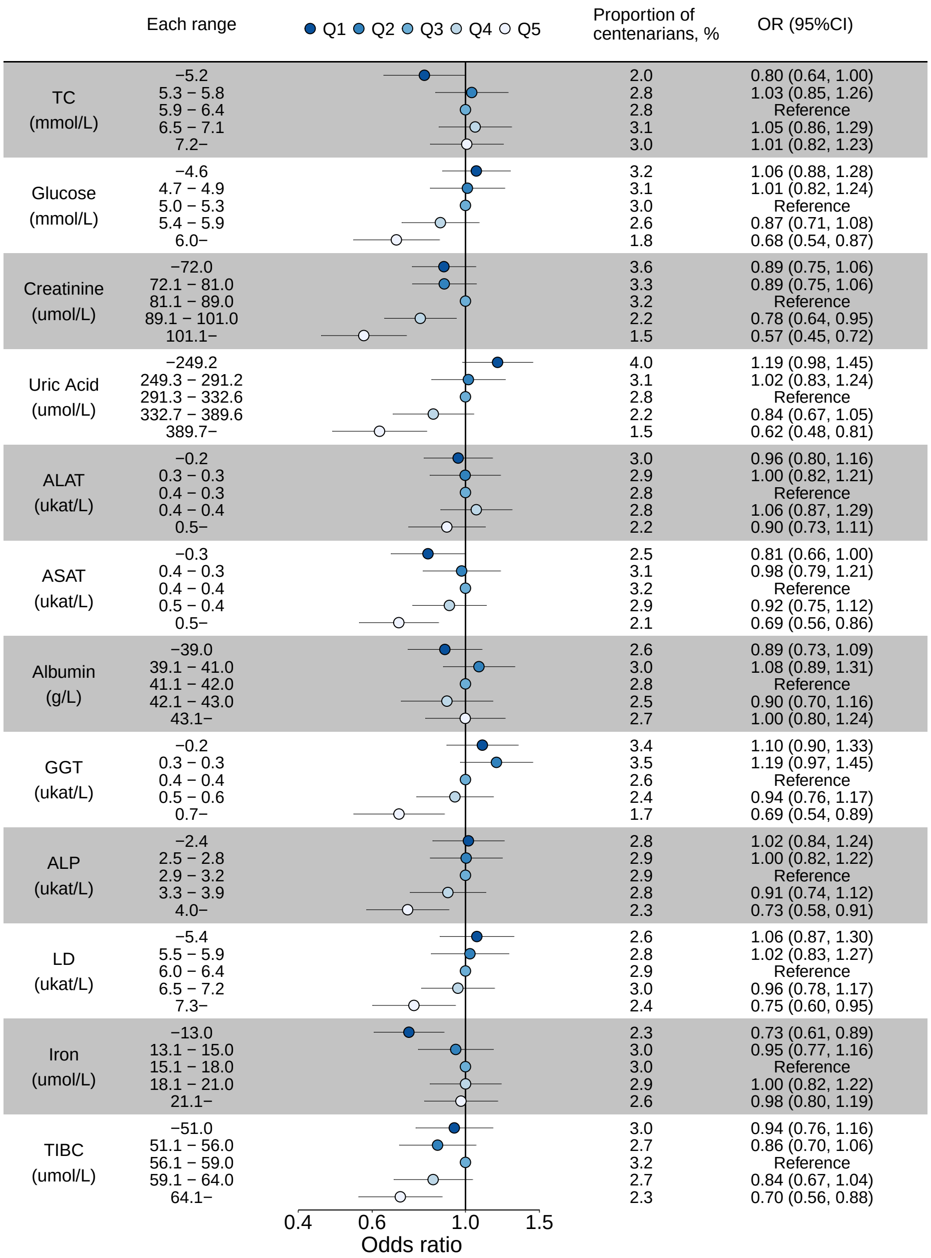

Supplemental figure 6: Association between biomarker quintiles and becoming a centenarian estimated with logistic regression adjusted for age, sex, and each comorbidities using multiply imputed data.

Multiply imputed data included 44,636 participants.

TC, total cholesterol; ALAT, alanine aminotransferase; ASAT, aspartate aminotransferase; GGT, gamma-glutamyl transferase; ALP, alkaline phosphatase; LD, lactate dehydrogenase; TIBC, total iron-binding capacity; CCI, Charlson comorbidity index; OR, odds ratio; CI, confidence interval

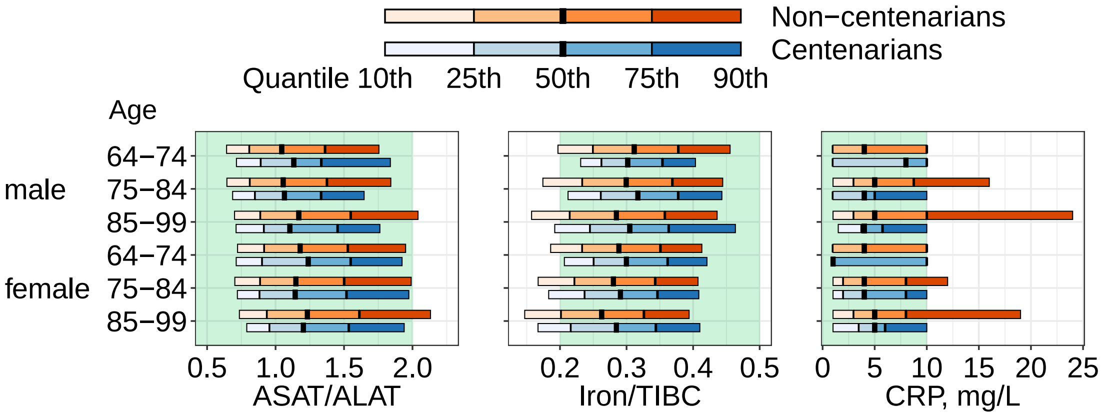

Supplemental figure 7: Quantiles (10th, 25th, 50th, 75th, 90th) of CRP, ASAT/ALAT, and iron/TIBC between centenarians and non-centenarians.

Green areas show each biomarker's normal range [7, 12, 17]. ALAT, alanine aminotransferase; ASAT, aspartate aminotransferase; TIBC, total iron-binding capacity; CRP, C-reactive protein. ASAT/ALAT and iron/TIBC were analyzed using multiply imputed data, CRP was analyzed using complete case data. 0.5% of participants excluded due to differences in assessment of ASAT and ALAT or iron and TIBC. CRP was measured in 46% (N=20,455) of participants, which were included in the analysis.

Supplemental table 6: Association of CRP, ASAT/ALAT, and iron/TIBC quintile with the chance of becoming a centenarian: logistic regression.

Biomarkers	Range	Proportion of centenarians	OR (95%CI)	*p*
ASAT/ALAT*				
Q1	- 0.82	2.3	0.94 (0.77, 1.16)	0.585
Q2	0.83 - 1.03	2.7	0.98 (0.81, 1.19)	0.852
Q3	1.04 - 1.25	2.8	Reference	
Q4	1.26 - 1.59	3.0	1.00 (0.82, 1.21)	0.990
Q5	1.60 -	2.8	0.89 (0.73, 1.08)	0.223
iron/TlBC*				
Q1	- 0.21	2.3	0.72 (0.57, 0.89)	0.003
Q2	0.22 - 0.27	2.7	0.89 (0.73, 1.09)	0.275
Q3	0.28 - 0.31	3.0	Reference	
Q4	0.32 - 0.37	3.0	1.07 (0.88, 1.31)	0.480
Q5	0.38 -	2.6	0.99 (0.80, 1.22)	0.932
CRP†				
Q1	1	3.0	1.09 (0.85, 1.41)	0.487
Q2	1.1 - 4	3.6	1.10 (0.86, 1.40)	0.466
Q3	4.1 - 6	3.2	Reference	
Q4	6.1 - 10	2.8	1.05 (1.81, 1.37)	0.692
Q5	10.1 -	1.8	0.62 (0.43, 0.89)	0.012

CRP, C-reactive protein; ALAT, alanine aminotransferase; ASAT, aspartate aminotransferase; TIBC, total iron-binding capacity; OR, odds ratio; CI, confidence interval

Each model was adjusted by age, sex, and CCI.

*ASAT/ALAT and iron/TIBC were analyzed using multiply imputed data. 0.5% of participants excluded due to difference in assessment of ASAT and ALAT or iron and TIBC.

†Complete case data were used when analyzing CRP. CRP was measured in 46% (N=20,455) of participants, which were included.

Supplemental table 7: Quantile regression comparing differences in 25th, 50th (median), 75th quantiles between clusters.

biomarkers	*p* of 25th quantile	*p* of 50th quantile	*p* of 75th quantile
TC	0.064	0.037*	0.048*
Glucose	0.220	0.243	0.351
Creatinine	0.694	0.594	0.517
Uric Acid	0.456	0.426	0.413
Albumin	0.028*	0.063	0.038*
GGT	0.355	0.370	0.258
ALP	0.508	0.734	0.773
Iron	0.696	0.749	0.721
TIBC	0.032*	0.0496*	0.0495*

TC, total cholesterol; GGT, gamma-glutamyl transferase;
ALP, alkaline phosphatase; TIBC, total iron-binding capacity
*p<0.05. Each model was adjusted with age and sex.
Multiply imputed data were used and 1,224 centenarians were included.

Detailed results of the clustering process and variable selection

Supplemental figure 9 shows detailed results of the procedure to select the number clusters and variables. In 68% of imputed data sets, 2 clusters were detected. The proportions of individuals in cluster 1 and 2 were 53.0% and 47.0%, respectively. The between-imputation distribution of CritCF also shows that selecting 2 clusters was appropriate. Most frequently, 6 variables were selected. ASAT, ALAT, and LD were never selected in 100 imputed data sets. Therefore, ASAT, ALAT, and LD were not used in further analyses.

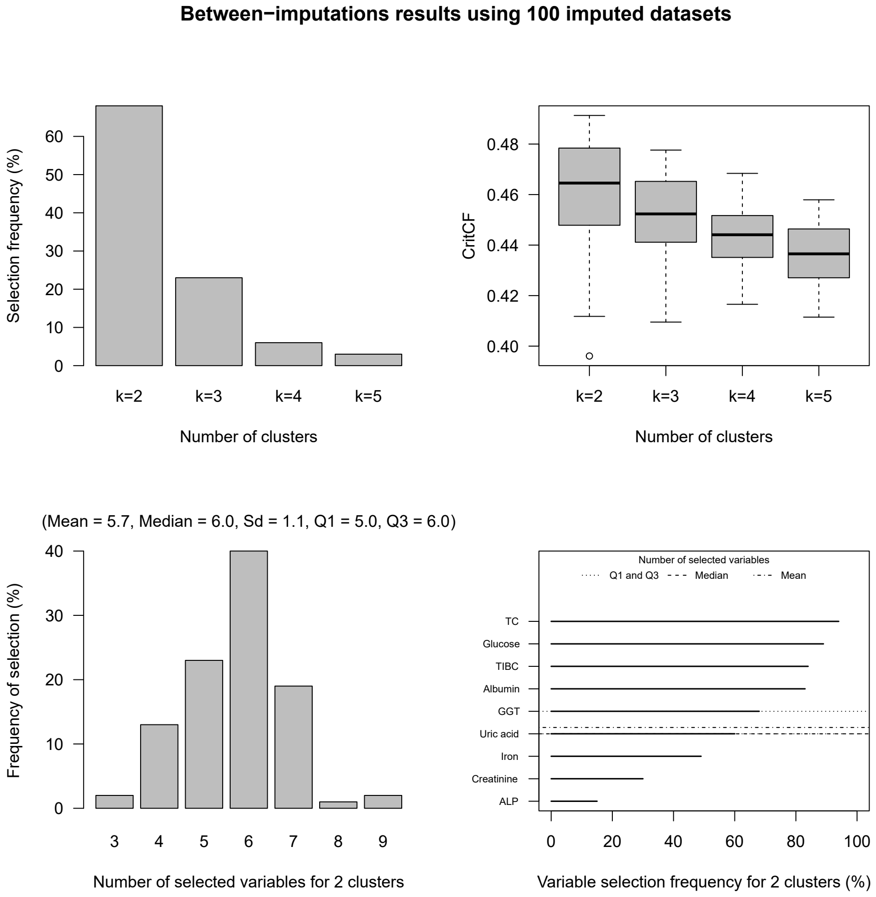

Supplemental figure 8: Detailed results of the K-medians clustering procedure using multiply imputed data. Upper left panel shows the detected number of clusters in 100 imputed data sets. Upper right panel shows box plots of the between-imputation distribution of CritCF by number of clusters. Lower left panel shows the distribution of the selected variable number in 100 imputed data sets. Lower right panel shows how frequently variables were selected in 2-cluster scenarios (k=2).

Supplemental table 8: Characteristics of individuals in each cluster.

	Higher nutrition (47.0%)	Lower but enough nutrition (53.0%)
Age, %		
-69	10.4	10.0
70-74	18.1	19.4
75-79	25.8	25.2
80-84	23.6	20.9
85-89	12.3	13.6
90-94	7.2	8.1
95-	2.6	2.8
Female, %	83.5	85.5
Charlson comorbidity index, %		
0	87.4	88.8
1	9.0	7.4
2 or more	3.6	3.7

Cluster 1 and 2 were named ”higher nutrition” and ”lower but enough nutrition”.

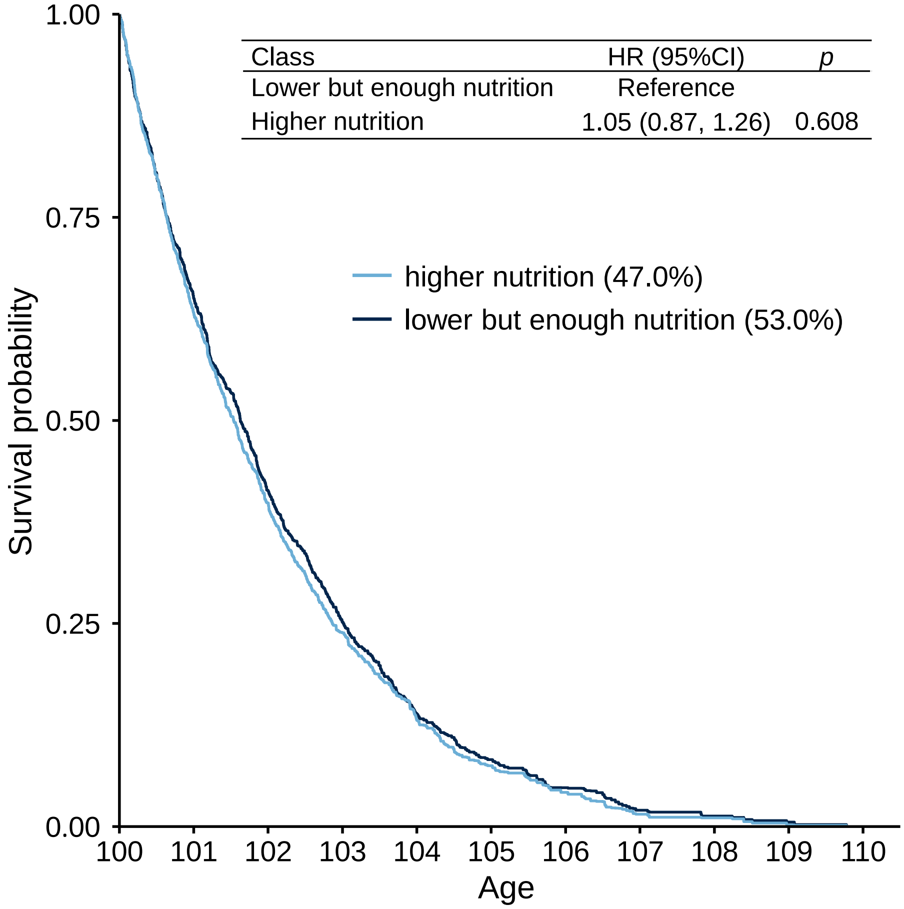

Supplemental figure 9: Comparison of suvival probability between clusters: Kaplan-Meier survival curves and Cox proprotional hazard model.

HR were estimated using a crude Cox model. Multiply imputed data were used and 1,224 centenarians were included. HR, hazard ratio; CI, confidence interval

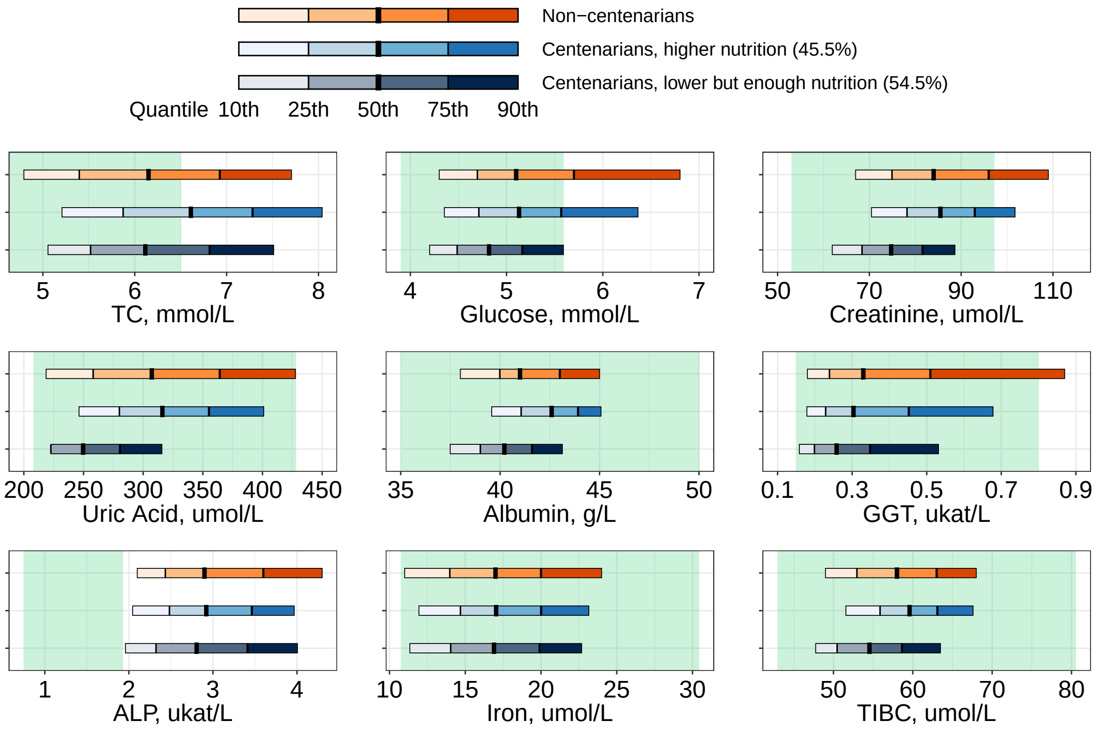

Supplemental figure 10: Quantiles (10th, 25th, 50th, 75th, 90th) of selected biomarkers among each centenarian cluster and non-centenarians restricting to participants whose blood was sampled before age 80 years.
Green areas show each biomarker's normal range. Multiply imputed data were used and 667 centenarians and 30,796 non-centenarians were included.
TC, total cholesterol; ALAT, alanine aminotransferase; ASAT, aspartate aminotransferase; GGT, gamma-glutamyl transferase; ALP, alkaline phosphatase; TIBC, total iron-binding capacity.

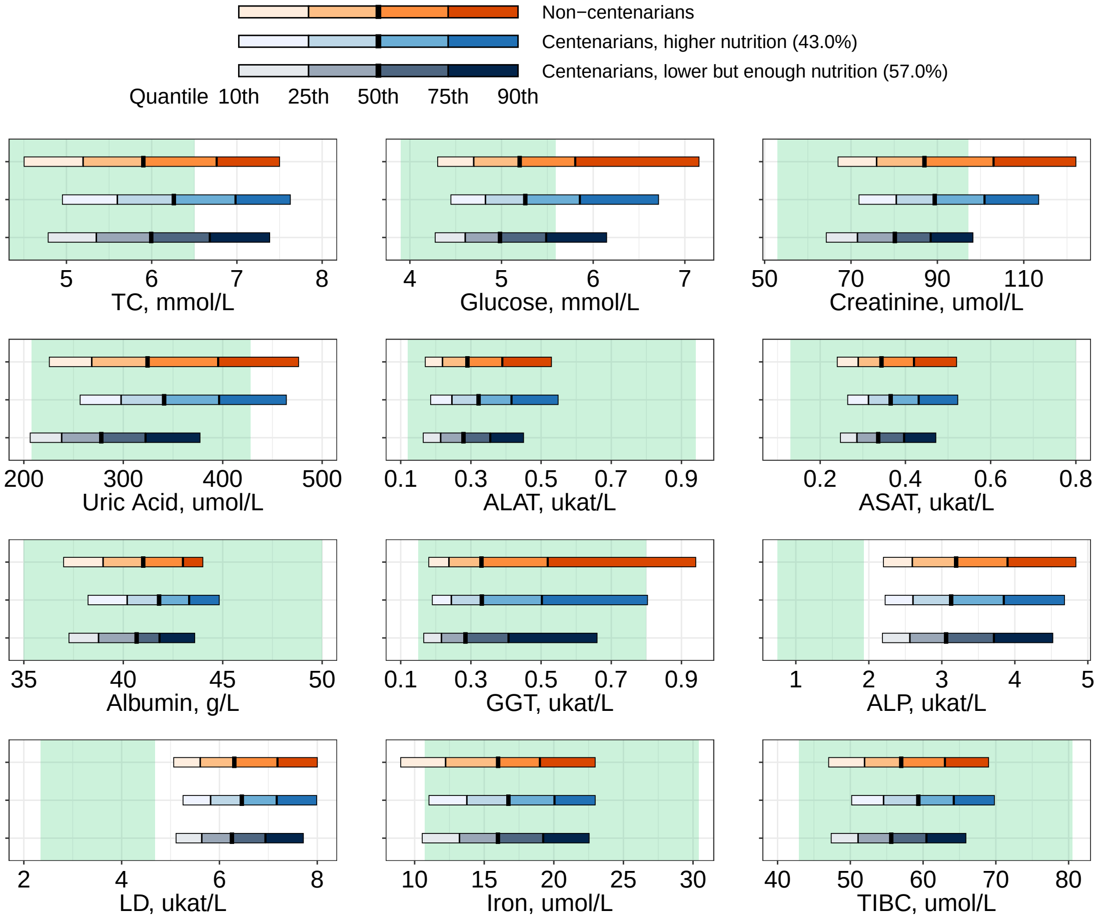

Supplemental figure 11: Quantiles (10th, 25th, 50th, 75th, 90th) of selected biomarkers among each centenarian cluster and non-centenarians restricting to participants whose blood was sampled at age 80 or older.

Green areas show each biomarker's normal range. Multiply imputed data were used and 557 centenarians and 12,616 non-centenarians were include.

TC, total cholesterol; ALAT, alanine aminotransferase; ASAT, aspartate aminotransferase; GGT, gamma-glutamyl transferase; ALP, alkaline phosphatase; LD, lactate dehydrogenase; TIBC, total iron-binding capacity

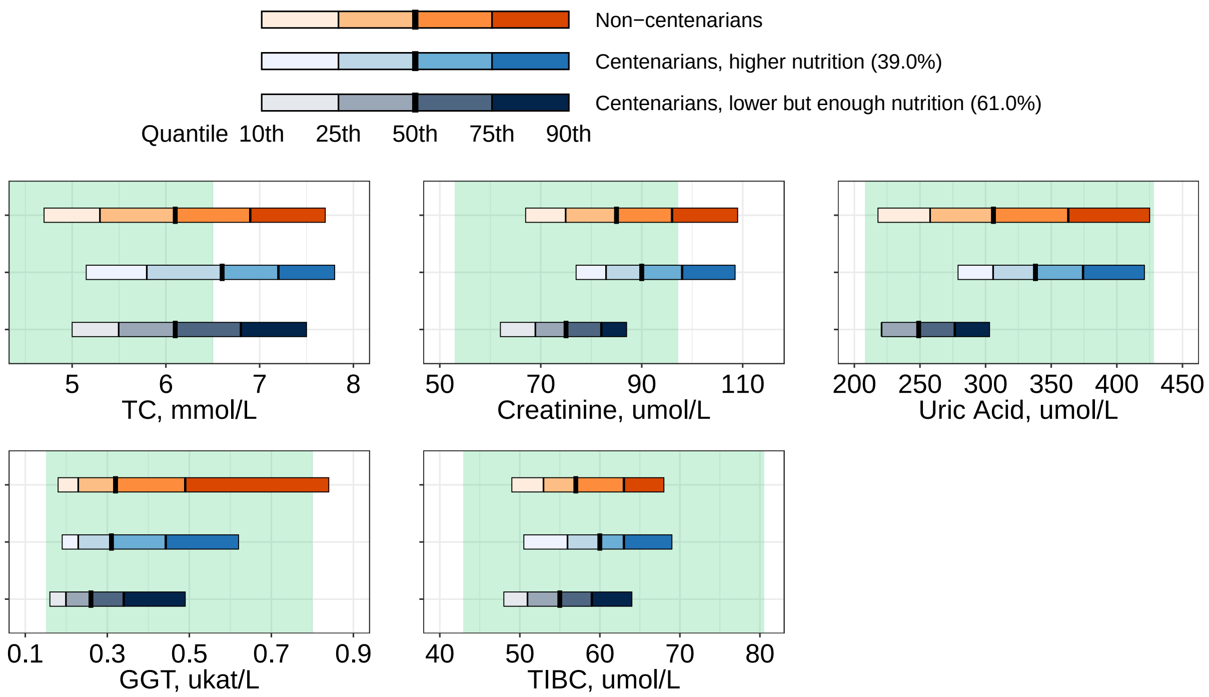

Supplemental figure 12: Quantiles (10th, 25th, 50th, 75th, 90th) of selected biomarkers among each centenarian cluster and non-centenarians using complete case data.

Green areas show each biomarker's normal range. Complete case data included 707 centenarians and 25,959 non-centenarians.

TC, total cholesterol; GGT, gamma-glutamyl transferase; TIBC, total iron-binding capacity

Reference

1. Jungner I, Walldius G, Holme I, Kolar W, Steiner E. Apolipoprotein B and A-I in relation to serum cholesterol and triglycerides in 43 000 Swedish males and females. *Int J Clin Lab Res* 1992; 21: 247-55.
2. American Diabetes Association. Diagnosis and Classification of Diabetes Mellitus. *Diabetes Care* 2013; 37: S81-S90.
3. Mount Sinai Hospital. Creatinine Blood Test. In: Dugdale III DC ZD, Conaway B, eds. https://www.mountsinai.org/health-library/tests/creatinine-blood-test. Accessed October 3, 2022.
4. UCSF Health. Uric acid - blood. https://www.ucsfhealth.org/en/medical-tests/uric-acid—-blood-. Accessed October 3, 2022.
5. Mayo Clinic. Tests and Procedures. Liver function tests. http://www.mayoclinic.org/tests-procedures/liver-function-tests/basics/results/prc-20012602. Accessed October 3, 2022.
6. US National Library of Medicine. Lactate dehydrogenase test. https://medlineplus.gov/ency/article/003471.htm. Accessed October 3, 2022.
7. Mount Sinai Hospital. Serum iron test. https://www.mountsinai.org/health-library/tests/serum-iron-test. Accessed October 3, 2022.
8. UCSF Health. Total iron binding capacity. https://www.ucsfhealth.org/medical-tests/total-iron-binding-capacity. Accessed October 3, 2022.
9. Shah AD, Bartlett JW, Carpenter J, Nicholas O, Hemingway H. Comparison of random forest and parametric imputation models for imputing missing data using MICE: a CALIBER study. Am J Epidemiol 2014; 179(6): 764-74.
10. Liaw A, Wiener M. Classification and regression by randomforest. R News 2 (3): 18–22. 2002.
11. Buuren Sv, Groothuis-Oudshoorn K. mice: Multivariate imputation by chained equations in R. Journal of statistical software 2010: 1-68.
12. Sorbi D, Boynton J, Lindor KD. The ratio of aspartate aminotransferase to alanine aminotransferase: potential value in differentiating nonalcoholic steatohepatitis from alcoholic liver disease. *Am J Gastroenterol*. 1999;94:1018-1022.
13. Cacoub P, Vandewalle C, Peoc'h K. Using transferrin saturation as a diagnostic criterion for iron deficiency: A systematic review. Crit Rev Clin Lab Sci 2019; 56: 526-32.
14. Lenth R, Singmann H, Love J, Buerkner P, Herve M. emmeans: estimated marginal means,

AKA least- squares means. R package version 1.7.2 2019.

15. Basagana X, Barrera-Gomez J, Benet M, Anto JM, Garcia-Aymerich J. A framework for multiple imputation in cluster analysis. *Am J Epidemiol*. 2013;177(7):718-725.

16. Aha DW, Bankert RL. A Comparative Evaluation of Sequential Feature Selection Algorithms. In: Fisher D, Lenz H-J, eds. Learning from Data: Artificial Intelligence and Statistics V. New York, NY: Springer New York; 1996:199-206.

17. Mayo Clinic. Tests and Procedures. C-reactive protein test. https://www.mayoclinic.org/tests-procedures/c-reactive-protein-test/about/pac-20385228. Accessed October 3, 2022.
